# Performance Bound for Extended Target Tracking Using High Resolution Sensors

**DOI:** 10.3390/s101211618

**Published:** 2010-12-20

**Authors:** Zhiwen Zhong, Huadong Meng, Hao Zhang, Xiqin Wang

**Affiliations:** Department of Electronic Engineering, Tsinghua University, Beijing 100084, China; E-Mails: zhiwenz@gmail.com (Z.Z.); haozhang@tsinghua.edu.cn (H.Z.); wangxq_ee@tsinghua.edu.cn (X.W.)

**Keywords:** posterior Cramer-Rao lower bound (PCRLB), Fisher information matrix (FIM), extended information reduction factor (EIRF), extended target tracking

## Abstract

This article concerns the problem of the estimation bound for tracking an extended target observed by a high resolution sensor. Two types of commonly used models for extended targets and the corresponding posterior Cramer-Rao lower bound (PCRLB) are discussed. The first type is the equation-extension model which extends the state space to include parameters such as target size and shape. Thus, the extended state vector can be estimated through the measurements obtained by a high resolution sensor. The measurement vector is also an expansion of the conventional one, and the additional measurements such as target extent can provide extra information for the estimation. The second model is based on multiple target measurements, each of which is an independent random draw from a spatial probability distribution. As the number of measurements per frame is unknown and random, the general form of the measurement contribution to the Fisher information matrix (FIM) conditional on the number of measurements is presented, and an extended information reduction factor (EIRF) approach is proposed to calculate the overall FIM and, therefore, the PCRLB. The bound of the second extended target model is also less than that of the point model, on condition that the average number of measurements is greater than one. Illustrative simulation examples of the two models are discussed and demonstrated.

## 1. Introduction

In a conventional target tracking framework, it is usually assumed that the sensor obtains one measurement of a single target (if detected) at each time step, which is referred to as the point target model. However, high resolution sensors have recently become more widely used and are able to resolve multiple point features on a single extended target. The potential to make use of the multiple sensor measurements is referred to as extended target tracking. An extended target is usually seen as a rigid or semi-rigid body. In contrast to the conventional point target model, the measurements provided by high-resolution sensors can provide extra information to improve target identification and data association [1,2]. Due to the complexity and nonlinearity of the extended target models, many kinds of nonlinear filtering algorithms using various assumptions have been developed [1–5]. However, the optimal solution of the filtering problem in extended target tracking is often unachievable. The posterior Cramer-Rao lower bound (PCRLB) [6] provides a mean square error bound on the performance of any unbiased estimator of an unknown stochastic parameter vector. In the context of target tracking, the PCRLB enables one to determine a bound on the optimal achievable accuracy of target state estimation.

The calculation of the PCRLB for two different types of extended target tracking models is considered in this paper. The first type of extended target tracking model extends the state and measurement equations [1,4,5,7]. Some features of the extended target, such as the target extent in one or more dimensions, are obtained from the multiple point features by the high resolution sensor. Parameters that may indicate the target size and shape can be added into the state vector and estimated through the extended dynamic and measurement equations [1,5,7]. In [7], the PCRLB of the target centroid dynamics of the extended target model was proven to be always smaller than that of the point target model under certain sufficient conditions. This conclusion suggests the use of the extended target model to potentially achieve better performance in tracking applications, and is generalized to the cluttered environments in [8].

In the second type of extended target tracking approaches, the state space is the same as that of the point target model, and the measurement of the target is represented by a spatial probability distribution. The target states are estimated based on the multiple measurements, which come from a region of high spatial density [3]. The measurements are usually independent and identically distributed variables, and the total number per frame is unknown and random. The recursive computation of the PCRLB adopted from [9] is then adjusted as a result of the uncertainty of the measurement origin. Referencing the ideas of calculating the PCRLB in cluttered environments (at most one measurement originated from the target per frame but with missed detections and false alarms) [10–14], the general form of the measurement contribution to the Fisher information matrix (FIM) is given, and the extended information reduction factor (EIRF) approach is introduced. The EIRF method averages the measurement contribution conditional on the number of sensor measurements to obtain an unconditional measurement contribution, and then the recursion of the PCRLB proceeds.

The paper is organized as follows. Section 2 introduces the definition and recursive formulation of the PCRLB for the general nonlinear filtering problem. Section 3 introduces the calculation and theoretical development of the PCRLB for two types of extended target tracking models. In Section 4, illustrative simulation examples corresponding to the two types of models are presented and discussed. Conclusions are given in Section 6.

## 2. Posterior Cramer-Rao Lower Bound

### 2.1. Definition

Let *X̄_k_* denote any unbiased estimator of the vector *X_k_* (unknown and random); the covariance of *X̄_k_* has a lower bound that is expressed as follows [6]:

(1) $$C_{k}=E\left[\left({\hat{X}}_{k}-X_{k}\right){\left({\hat{X}}_{k}-X_{k}\right)}^{T}\right]\geq J_{k}^{-1}$$

where *J_k_* is referred to as the Fisher information matrix (FIM). The inverse of the FIM $J_{k}^{-1}$ is the PCRLB. The inequality in (1) means that the difference $C_{k}-J_{k}^{-1}$ is a positive semi-definite matrix.

### 2.2. Recursive Form of the PCRLB

Tichavsky *et al*. [9] provided a Riccati-like recursion to calculate the FIM *J_k_* for the general nonlinear filtering problem. The parameters to be estimated are contained in the state vector *X_k_*, where *k* denotes the time step. At each time step, the sensor obtains one measurement vector *Z_k_*. The general form of the dynamic and measurement model is:

(2) $$X_{k+1}=f_{k}\left(X_{k},q_{k}\right)$$

(3) $$z_{k}=h_{k}\left(X_{k},r_{k}\right)$$

where *f_k_*( ) and *h_k_*( ) are (in general) nonlinear functions, and *q_k_* and *r_k_* are the dynamic and measurement noise, respectively, which are assumed to be independent and white processes (*i.e.*, sequences of mutually independent random variables or vectors). The FIM is then computed recursively as:

(4) $$J_{k+1}=D_{k}^{33}-D_{k}^{21}{\left(J_{k}+D_{k}^{11}\right)}^{-1}D_{k}^{12}+J_{Z}\left(k+1\right)$$

where:

(5) $$D_{k}^{11}=E\left\{-\Delta_{x_{k}}^{x_{k}}\text{ln}\;p\left(X_{k+1}|X_{k}\right)\right\}$$

(6) $$D_{k}^{12}=E\left\{-\Delta_{x_{k}}^{x_{k+1}}\text{ln}\;p\left(X_{k+1}|X_{k}\right)\right\}$$

(7) $$D_{k}^{33}=E\left\{-\Delta_{x_{k+1}}^{x_{k+1}}\text{ln}\;p\left(X_{k+1}|X_{k}\right)\right\}$$

(8) $$J_{Z}\left(k+1\right)=E\left\{{-\Delta }_{x_{k+1}}^{x_{k+1}}\text{ln}\;p\left(z_{k+1}|X_{k+1}\right)\right\}$$

and $\Delta_{\phi }^{\theta }$ is a second-order partial derivative operator defined as:

(9) $$\Delta_{\phi }^{\theta }=\nabla_{\phi }\nabla_{\theta }^{T}$$

where:

(10) $$\nabla_{\phi }={\left[\frac{\partial }{\delta \phi_{1}},\ldots ,\frac{\partial }{\delta \phi_{n}}\right]}^{T}$$

is a first-order partial derivative operator with respect to the parameter vector *φ*. Throughout this article *E_φ_* will denote mathematical expectation with respect to *φ*. In Equation (4)*J_z_* (*k* + 1) denotes the measurement contribution to the FIM. The PCRLB is then the inverse of the FIM calculated recursively through Equation (4) and the initial target distribution covariance *C*_0_ (the initial FIM is the inverse of the initial target distribution covariance, *i.e.*, $J_{0}=C_{0}^{-1}$).

If the dynamic and measurement noise are additive Gaussian, *i.e.*:

(11) $$X_{k+1}=f_{k}\left(X_{k}\right)+q_{k},\;\;\;q_{k}∼N\left(0,Q_{k}\right)$$

(12) $$z_{k}=h_{k}\left(X_{k}\right)+r_{k},\;\;\;\;r_{k}∼N\left(0,R_{k}\right)$$

where *N* (*μ*,Σ) denotes the Gaussian distribution with mean *μ* and covariance Σ, the recursive formula is simplified to:

(13) $$J_{k+1}=J_{Z}\left(k+1\right)+Q_{k}^{-1}-Q_{k}^{-1}E\left[F_{k}\right]{\left(J_{k}+E\left[F_{k}^{T}Q_{k}^{-1}F_{k}\right]\right)}^{-1}E\left[F_{k}^{T}\right]Q_{k}^{-1}$$

and:

(14) $$J_{z}\left(k+1\right)=E_{X_{k+1}}\left[H_{k+1}^{T}R_{k+1}^{-1}H_{k+1}\right]$$

where *F_k_* and *H_k_* are the Jacobians of the nonlinear functions *f_k_* (*x_k_*) and *h_k_* (*x_k_*), *i.e.*:

(15) $$F_{k}={\left[\nabla_{x_{k}}f_{k}^{T}\left(x_{k}\right)\right]}^{T}$$

(16) $$H_{k}={\left[\nabla_{x_{k}}h_{k}^{T}\left(x_{k}\right)\right]}^{T}$$

Furthermore, if the target dynamics are linear (*i.e.*, *f_k_* (*x_k_*) = *A_k_X_k_*), then it is straightforward to show that:

(17) $$J_{k+1}={\left(Q_{k}+A_{k}J_{k}^{-1}A_{k}^{T}\right)}^{-1}+J_{Z}\left(k+1\right)$$

where *J_z_* (*k* + 1) is given in (14). Equation (17) also holds if the target dynamics are non-random (*i.e.*, *Q_k_* = 0).

## 3. PCRLBs for Extended Target Tracking

### 3.1. The Equation Extension Model

For the first type of extended target tracking models, the dynamic and measurement vectors are both extensions of the ones in the point model with additional states and measurements. The equations are thus also extensions of the point model and are still in the general form. The superscripts “*p*” and “*e*” are used in the paper to indicate the “point” and “extended” target tracking models, respectively. Thus, the extended model is expressed as:

(18) $$X_{k+1}^{e}=f_{k}^{e}\left(X_{k}^{e},q_{k}^{e}\right)$$

(19) $$z_{k}^{e}=h_{k}^{e}\left(X_{k}^{e},r_{k}^{e}\right)$$

where $X^{e}=\left[\begin{array}{l} X^{p}\\ X^{n} \end{array}\right]$ and the parameters of the extended target, such as target size and shape, are expressed by the additional state vector *X^n^*. The PCRLB can then be calculated through the general recursive equation in (4). As proven in [7], under certain sufficient conditions, which are satisfied in most extended target tracking applications, the bound of the target centroid dynamics is always smaller than that of the point model:

(20) $$\Gamma \left[{\left(J_{k}^{e}\right)}^{-1}\right]<{\left(J_{k}^{p}\right)}^{-1}$$

where Γ (·) is a function to obtain the $N_{S}^{p}\times N_{S}^{p}$ left-upper sub-matrix and $N_{S}^{p}$ is the dimensionality of the state vector *X^p^*. Furthermore, the conclusion still holds in cluttered environments [8].

### 3.2. The Spatial Probability Distribution Model—The Extended Information Reduction Factor (EIRF) Approach

In the second type of extended target tracking framework, the state space and the dynamic equation of the extended target model are in the general form described by (2). However, a high resolution sensor can obtain multiple measurements at each time step. It is assumed that *m_k_* independent measurements generated from the target, denoted as Z*_k_* ≜ {Z*_k_* (*i*):*i* = 1,2,…,m*_k_*}, are observed by the sensor. Each of the measurements is distributed according to the known spatial extent model *p_z_* (*Z_k_* (*i*) | *X_k_*), also in the measurement equation form:

(21) $$Z_{k}(i)=h_{k}\left(X_{k},r_{k}\left(i\right)\right),\;\;\;i=1,\ldots ,m_{k}$$

where *r_k_* (*i*) are *i.i.d.* stochastic vectors and the total number of measurements *m_k_* is unknown and random. The overall probability density is then:

(22) $$p\left(Z_{k}|X_{k},m_{k}\right)=\prod_{i=1}^{m_{k}}p_{z}\left(Z_{k}\left(i\right)|X_{k}\right)$$

For the spatial distribution model of the extended target, the number of sensor measurements per frame is unknown and random. Thus, the recursive calculation of the PCRLB cannot be applied directly. Referencing the ideas for calculating the PCRLB in the case of a single point target in a cluttered environment [10,12,13], the general form of the measurement contribution is given, and an extended information reduction factor (EIRF) approach is proposed to calculate the PCRLB. The measurement contribution at time *k* given that there are *m_k_* measurements at that time is thus:

(23) $$\begin{array}{ll} J_{Z}\left(k:m_{k}\right) & =E\left\{-\Delta_{X_{k}}^{X_{k}}\;\text{ln}\;p\left(Z_{k}|X_{k},m_{k}\right)\right\}\\ & =E\left\{-\Delta_{X_{k}}^{X_{k}}\;\text{ln}\;\left[\prod_{i=1}^{m_{k}}p_{z}\left(Z_{k}\left(i\right)|X_{k}\right)\right]\right\}\\ & =\sum_{i=1}^{m_{k}}E\left\{-\Delta_{X_{k}}^{X_{k}}\;\text{ln}\;p_{z}\left(Z_{k}\left(i\right)|X_{k}\right)\right\}. \end{array}$$

It is noticed that for each *i* (1 ≤ *i* ≤ *m_k_*), *Z_k_* (*i*) is *i.i.d.* to *p_z_* (*z_k_* | *X_k_*), then:

(24) $$E\left\{-\Delta_{X_{k}}^{X_{k}}\text{ln}\;p_{z}\left(Z_{k}\left(i\right)|X_{k}\right)\right\}=E\left\{-\Delta_{X_{k}}^{X_{k}}\;\text{ln}\;p_{z}\left(z_{k}|X_{k}\right)\right\}$$

for all *i* (1 ≤ *i* ≤ *m_k_*). It then follows from (23) and (24) that:

(25) $$J_{Z}\left(k:m_{k}\right)=m_{k}E\left\{-\Delta_{X_{k}}^{X_{k}}\;\text{ln}\;p_{z}\left(z_{k}|X_{k}\right)\right\}.$$

The following expression is defined as the measurement contribution in the conventional case (where just one measurement originates from *p_z_* (*z_k_* | *X_k_*) at time *k*):

(26) $$J_{Z1}\left(k\right)=E\left\{-\Delta_{X_{k}}^{X_{k}}\;\text{ln}\;p_{z}\left(z_{k}|X_{k}\right)\right\}$$

Thus:

(27) $$J_{Z}\left(k:m_{k}\right)=m_{k}J_{Z1}\left(k\right)$$

The conclusion above obeys the usual intuition that the measurement uncertainty is reduced by multiple *i.i.d.* measurements (for the case that *m_k_* > 1).

If the dynamic and measurement noise are additive Gaussian (described by (11) and (12)), the measurement contribution is then written as follows [using (14) and (27)]:

(28) $$J_{Z}\left(k:m_{k}\right)=m_{k}E_{X_{k}}\left[H_{k}^{T}R_{k}^{-1}H_{k}\right]$$

Equation (25) indicates that the measurement uncertainty brought by multiple target generated measurements is generalized to a single multiplier that equals to the number of measurements at the corresponding time. The overall measurement contribution can then be calculated as a weighted sum of the conditional ones, which is referred to as the extended information reduction factor (EIRF) approach. The overall measurement contribution is formulated as:

(29) $$\begin{array}{ll} J_{Z}(k) & =E_{m_{k}}\left[J_{Z}\left(k:m_{k}\right)\right]\\ & =\sum_{m_{k}}p\left(m_{k}\right)J_{Z}\left(k:m_{k}\right)\\ & =\sum_{m_{k}}p\left(m_{k}\right)\cdot m_{k}\cdot J_{Z1}\left(k\right)\\ & =E\left[m_{k}\right]\cdot J_{Z1}\left(k\right)\\ & =E\left[m_{k}\right]\cdot E\left\{-\Delta_{X_{k}}^{X_{k}}\;\text{ln}\;p_{z}\left(z_{k}|X_{k}\right)\right\} \end{array}$$

where *p*(*m_k_*) is the probability that *m_k_* measurements are obtained by the sensor and *E*[*m_k_*] is the mathematical expectation of the number of measurements at time step *k*. Note that the EIRF (which is equal to *E*[*m_k_*]) can be either greater or less than one. Hence, the information might be either reduced or enlarged. For a common extended target tracking scenario, the mean number of measurements is usually much greater than one, so that the information is enlarged and therefore the PCRLB is decreased. Hence the estimation performance might be improved through the multiple sensor measurements.

Because the probability distribution of *m_k_* is usually prior information in a specific sensor and target scenario, the value of the measurement contribution can easily be calculated using (29). Then, the FIM *J_k_* can be calculated through the recursion in (4) together with the initial FIM $J_{0}=C_{0}^{-1}$. The corresponding EIRF bound is denoted as:

(30) $$\text{PCRLB}(\text{EIRF};k)=J_{k}^{-1}$$

If the dynamic and measurement noise are additive Gaussian (described by (11) and (12)), the overall measurement contribution is then:

(31) $$J_{Z}(k)=E\left[m_{k}\right]E_{X_{k}}\left[H_{k}^{T}R_{k}^{-1}H_{k}\right]$$

Furthermore, if the target dynamics are linear (*i.e.*, *f_k_* (*x_k_*) = *A_k_X_k_*), the recursion of *J_k_* is then simplified to (17), with *J_z_* (*k*) calculated by (31).

## 4. Illustrative Simulation Examples

The following simulation examples are presented to illustrate the numerical results of PCRLBs for the two types of extended target tracking models.

### 4.1. Example 1: Stick Shaped Extended Target Tracking

**Figure 1.**
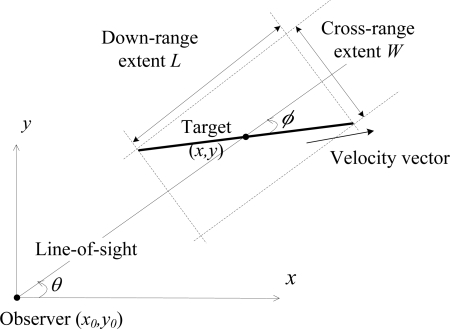
The stick shaped extended target model.

The first example is tracking an extended target whose shape is modeled as a stick using an equation-extension model for extended target tracking, which is described in Section 3.1. As shown in Figure 1, the sensor is located at (*x*_0_, *y*_0_) on a 2-D plane, and the state vector of the target is *X^e^* = (*x*, *ẋ*, *y*, *ẏ*, *l*), where *l* is the target length and the superscript “*e*” denotes the extended model. The target is moving with nearly constant velocity (NCV), and the direction of the velocity is assumed to be along the stick. The dynamic model is:

(32) $$X_{k+1}^{e}=F_{k}^{e}X_{k}^{e}+q_{k}^{e}=\left[\begin{array}{lllll} 1 & T & 0 & 0 & 0\\ 0 & 1 & 0 & 0 & 0\\ 0 & 0 & 1 & T & 0\\ 0 & 0 & 0 & 1 & 0\\ 0 & 0 & 0 & 0 & 1 \end{array}\right]X_{k}^{e}+q_{k}^{e}$$

where *T* is the time interval between sensor measurements and $q_{k}^{e}$ is the Gaussian dynamic noise with zero mean and covariance $Q_{k}^{e}$.

The sensor obtains not only the conventional target position measurements, such as the distance and azimuth angle of the target centroid, but also the extended measurements that describe the target extent. The measurement model is:

(33) $$z_{k}^{e}=h_{k}^{e}\left(X_{k}^{e}\right)+r_{k}^{e}=\left[\begin{array}{l} \rho \left(X_{k}^{e}\right)\\ \theta \left(X_{k}^{e}\right)\\ L\left(X_{k}^{e}\right)\\ W\left(X_{k}^{e}\right) \end{array}\right]+r_{k}^{p}$$

where $\left[\begin{array}{l} \rho \left(X^{e}\right)\\ \theta \left(X^{e}\right) \end{array}\right]=\left[\begin{array}{c} \sqrt{{\left(x-x_{0}\right)}^{2}+{\left(y-y_{0}\right)}^{2}}\\ \text{arctan}\frac{y-y_{0}}{x-x_{0}} \end{array}\right]$ is the conventional part of the measurement vector, denoting the distance and azimuth angle of the target centroid, and $\left[\begin{array}{c} L\left(X^{e}\right)\\ W\left(X^{e}\right) \end{array}\right]=\left[\begin{array}{c} l\text{cos}\varphi \\ l\text{sin}\varphi \end{array}\right]$ is the extended part of the measurement vector, denoting the down-range and cross-range extent of the target. The parameter is the angle between the line of sight (LOS) and the target velocity vector, denoted as the VLOS angle:

(34) $$\varphi =\text{arctan}\left[\frac{\dot{x}\left(y-y_{0}\right)-\dot{y}\left(x-x_{0}\right)}{\dot{x}\left(x-x_{0}\right)+\dot{y}\left(y-y_{0}\right)}\right].$$

The recursive computation of the FIM (13) can be applied here directly with the calculation of the measurement contribution in (14). The Jacobian $H_{k}^{e}$ in (14) is a 4 × 6 matrix. The non-zero elements of the first two rows are:

(35) $$H_{k}^{e}\left[1,1\right]=\frac{X_{k}^{e}\left(1\right)}{\sqrt{{\left[X_{k}^{e}\left(1\right)\right]}^{2}+{\left[X_{k}^{e}\left(3\right)\right]}^{2}}},\;\;\;\;H_{k}^{e}\left[1,3\right]=\frac{X_{k}^{e}\left(3\right)}{\sqrt{{\left[X_{k}^{e}\left(1\right)\right]}^{2}+{\left[X_{k}^{e}\left(3\right)\right]}^{2}}}$$

(36) $$H_{k}^{e}\left[2,1\right]=\frac{-X_{k}^{e}\left(3\right)}{{\left[X_{k}^{e}\left(1\right)\right]}^{2}+{\left[X_{k}^{e}\left(3\right)\right]}^{2}},\;\;H_{k}^{e}\left[2,3\right]=\frac{X_{k}^{e}\left(1\right)}{{\left[X_{k}^{e}\left(1\right)\right]}^{2}+{\left[X_{k}^{e}\left(3\right)\right]}^{2}}$$

The first four elements of the third and fourth row of the Jacobian $H_{k}^{e}$ are calculated by:

(37) $$\begin{array}{l} H_{k}^{e}\left(3,j\right)=\frac{\partial L\left(X_{k}^{e}\right)}{\partial X_{k}^{e}\left(j\right)}=-X_{k}^{e}\left(5\right)\cdot \text{sin}\varphi_{k}\cdot \frac{\partial \varphi_{k}}{\partial X_{k}^{e}\left(j\right)},\\ H_{k}^{e}\left(4,j\right)=\frac{\partial W\left(X_{k}^{e}\right)}{\partial X_{k}^{e}\left(j\right)}=X_{k}^{e}\left(5\right)\cdot \text{cos}\varphi_{k}\cdot \frac{\partial \varphi_{k}}{\partial X_{k}^{e}\left(j\right)},\;\;j=1,\ldots ,4 \end{array}$$

and $\frac{\partial \varphi_{k}}{\partial X_{k}^{e}\left(j\right)}$ can be calculated through (34):

(38) $$\left[\begin{array}{l} \frac{\partial \varphi_{k}}{\partial X_{k}^{e}\left(1\right)}\\ \frac{\partial \varphi_{k}}{\partial X_{k}^{e}\left(2\right)}\\ \frac{\partial \varphi_{k}}{\partial X_{k}^{e}\left(3\right)}\\ \frac{\partial \varphi_{k}}{\partial X_{k}^{e}\left(4\right)} \end{array}\right]=\left[\begin{array}{l} \frac{X_{k}^{e}(3)}{{\left[X_{k}^{e}(1)\right]}^{2}+{\left[X_{k}^{e}(3)\right]}^{2}}\\ \frac{-X_{k}^{e}(4)}{{\left[X_{k}^{e}(2)\right]}^{2}+{\left[X_{k}^{e}(4)\right]}^{2}}\\ \frac{-X_{k}^{e}(1)}{{\left[X_{k}^{e}(1)\right]}^{2}+{\left[X_{k}^{e}(3)\right]}^{2}}\\ \frac{X_{k}^{e}(2)}{{\left[X_{k}^{e}(2)\right]}^{2}+{\left[X_{k}^{e}(4)\right]}^{2}} \end{array}\right]$$

The last elements of the third and fourth row of the Jacobian $H_{k}^{e}$ are:

(39) $$\left[\begin{array}{l} H_{k}^{e}\left(3,5\right)\\ H_{k}^{e}\left(4,5\right) \end{array}\right]=\left[\begin{array}{l} \frac{\partial L\left(X_{k}^{e}\right)}{\partial X_{k}^{e}\left(5\right)}\\ \frac{\partial W\left(X_{k}^{e}\right)}{\partial X_{k}^{e}\left(5\right)} \end{array}\right]=\left[\begin{array}{l} \text{cos}\;\varphi_{k}\\ \text{sin}\;\varphi_{k} \end{array}\right]$$

In this simulation example, the sensor is static and located at the origin of the coordinate system, *i.e.*, (*x*_0_, *y*_0_) = (0, 0). The target moves with initial velocity *v*_0_ = 10 in the direction with the initial VLOS angle *Ø*_0_ = 20°. The initial position of the target is (15,000, 10,000), and the initial length is *l*_0_ = 50. The initial FIM is *J*_0_ = {[*diag* (20,3,20,3,1)]^2^}^−1^, and the covariance of the state noise is *Q_k_* = *diag* (3, 0.1, 3, 0.1, 1). The time interval between sensor measurements is *T* = 1, and the measurement noise is zero-mean white Gaussian noise with standard deviations: *σ_ρ_* = 5, *σ_θ_* = 0.1°, *σ_L_* = 3, and *σ_W_* = 3. All the parameter units are in the metric system.

**Figure 2.**
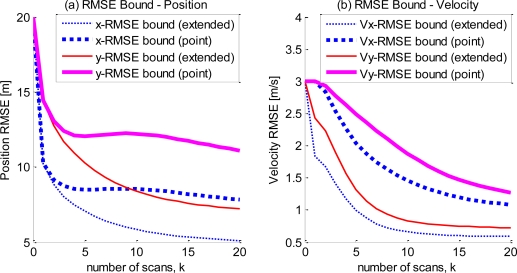
The comparison of the $\sqrt{\text{PCRLB}}$ curves of the extended and point target models for (a) the target position in the directions of the *x* and *y* axes and (b) the target velocity in the directions of the *x* and *y* axes.

Because the target dynamics are random (with non-zero dynamic noise) in the simulation scenario, the calculation of the measurement contribution using (14) requires the evaluation of the mathematical expectation of $H_{k+1}^{T}R_{k+1}^{-1}H_{k+1}$ with respect to *X_k_*_+1_. A sampling scheme is used here. From the initial target state and the dynamic model, multiple target state sequences are generated, and the corresponding measurement contribution is computed. The overall measurement contribution is then computed as an average of the measurement contributions conditional on each state sequence. In this simulation, 10,000 state sequences were sampled to approximate the measurement contribution. The comparison of the bounds of target centroid dynamics (position and velocity) using both the extended and point target models is shown in Figure 2. The numerical result also confirms the theoretical development in [7] that the bound of the extended model is always smaller than that of the point model because the three sufficient conditions proposed in [7] are satisfied in the tracking models discussed here. The improvement in the bound of the extended target tracking model is a result of the fact that the measurements of the target extent are directly dependent on the target centroid dynamics (position and velocity). Figure 2(a) shows that the new measurements of the target extent are of relatively small importance for the estimation of target position, especially in the early stage of tracking. However, the PCRLBs of the velocities [as shown in Figure 2(b)] of the extended target model in both the *x* and *y* directions decrease sharply upon the arrival of the measurements (the initial values of the FIM of the extended and point models are equivalent). In the conventional point target tracking framework, the velocity is estimated only through the target centroid positions, and the uncertainty of the velocity is not reduced until the second frame of measurements (so the bound of the velocity in the first scan is no less than the initial covariance). However, the information on the target velocity is carried with the target extent measurements in the extended target model. Thus, the uncertainty of the velocity is significantly reduced with the arrival of the first scan of sensor measurements. This advantage could greatly benefit the tracking systems for defense, especially for velocity-sensitive ones, such as anti-missile systems. The early and precise sensing of the target velocity will substantially improve the power of defense surveillance systems.

**Figure 3.**
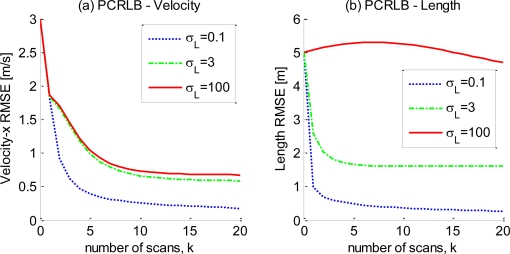
The comparison of the $\sqrt{\text{PCRLB}}$ curves for different values of σ*_L_* for (a) the target velocity in the direction of the *x* axis and (b) the target length.

Figure 3 illustrates the PCRLBs of the target velocity (in the direction of the *x* axis) and target length for different values of *σ_L_* (the effect of *σ_L_* on the $\sqrt{\text{PCRLB}}$ curves for the target position is negligible and therefore not shown here). In Figure 3, the parameters except *σ_L_* are set to the same values as before, and the curves of $\sqrt{\text{PCRLB}}$ corresponding to *σ_L_* = 0.1, *σ_L_* = 3, and *σ_L_* = 100 are presented. The numerical result unsurprisingly coincides with the intuition that the performance bound is improved by the accurate measurement of the target extent. Furthermore, Figure 3(b) shows that the estimation bound of the target length benefits from the accuracy of the target extent measurement more evidently than that of the target velocity [which is shown in Figure 3(a)] because the relationship between the target length and extent is more direct in this sensor-target geometry (*Ø*_0_ = 20°). The influence of the sensor-target geometry on the estimation bound is reported below.

**Figure 4.**
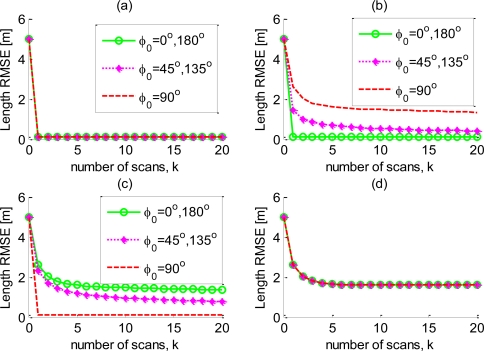
The impact of φ_0_ on the $\sqrt{\text{PCRLB}}$ curves for different values of σ*_L_* and σ*_W_* : (a) σ*_L_* = 0.1, σ*_W_* = 0.1. (b) σ*_L_* = 0.1, σ*_W_* = 3. (c) σ*_L_* = 3, σ*_W_* = 0.1. (d) σ*_L_* = 3, σ*_W_* = 3.

Next, the impact of the VLOS angle on the PCRLB is analyzed. The effect of *φ* on the estimation bounds of the position and velocity are negligible and not shown, but the impact of *φ* on the $\sqrt{\text{PCRLB}}$ curves for the target length also depends on the accuracy of the target extent measurements. Figure 4 shows the $\sqrt{\text{PCRLB}}$ curves for the initial VLOS angles *Ø*_0_ = 0°, 45°, and 90° for four combinations of the values of *σ_L_* and *σ_W_* (corresponding to the case of good and poor accuracy in the measurement of the down-range/cross-range extent).

In Figure 4(a) (down-range/cross-range extent are both accurate) and Figure 4(d) (down-range/cross-range extent are both inaccurate), the bounds corresponding to all initial VLOS angles are equivalent because the information provided by the down-range/cross-range extent is symmetric. In Figure 4(b), the bound for *Ø*_0_ = 0° (180°), is dramatically smaller than that for *Ø*_0_ = 90°. The superior performance bound is a result of the target orientation being along the direction with the best measurement accuracy.

A similar interpretation can be proposed for Figure 4(c). From this result, it follows that, for a moving sensor platform, it is possible to design an optimal movement trajectory that minimizes the PCRLB for the target length. When the down-range extent is more accurate than the cross-range extent, the optimal trajectory should make the VLOS angle approach 0°/180°; when the down-range extent is less accurate than the cross-range extent, it should approach 90°.

### 4.2. Example 2: The Multiple Measurement Extended Target Tracking

The following simulation example is presented to illustrate the performance bounds for tracking an extended target that can be resolved as multiple point features at each sampling time by a high resolution sensor. The simulation scenario discussed below is similar to Example 1 discussed in Section 5.1. The observer is located at (*x*_0_, *y*_0_) on a 2-D plane. The target is moving with nearly constant velocity (NCV), and the dynamic model is of the conventional form:

(40) $$X_{k+1}=A_{k}X_{k}+q_{k},\;q_{k}∼N\left(0,Q_{k}\right)$$

where *X* = (*x*, *ẋ*, *y*, *ẏ*)*^T^*, $A_{k}=\text{diag}\left(\left[\begin{array}{cc} 1 & T\\ 0 & 1 \end{array}\right],\left[\begin{array}{cc} 1 & T\\ 0 & 1 \end{array}\right]\right)$ and *T* is the time interval between measurements. The measurement model is:

(41) $$Z_{k}\left(i\right)=h_{k}\left(X_{k}\right)+r_{k}\left(i\right),\;\;\;\;r_{k}\left(i\right)∼N\left(0,R_{k}\right),\;\;\;i=1,\ldots ,m_{k}$$

where *m_k_* is the number of measurements, and:

(42) $$h_{k}\left(X_{k}\right)=\left[\begin{array}{l} \rho \left(X_{k}\right)\\ \theta \left(X_{k}\right) \end{array}\right]=\left[\begin{array}{c} \sqrt{{\left(x_{k}-x_{0}\right)}^{2}+{\left(y_{k}-y_{0}\right)}^{2}}\\ \text{arctan}\frac{y_{k}-y_{0}}{x_{k}-x_{0}} \end{array}\right]$$

includes the distance and azimuth angle of the target. As described in Section 2.2, *r_k_* (*i*) are *i.i.d.* stochastic vectors. The number of target measurements per frame *m_k_* is Poisson distributed, and the mean number is *λ_T_*. Thus, the probability that *m_k_* measurements originating from the target are observed is:

(43) $$\text{Pr}\left(m_{k}\right)=\frac{\lambda_{T}^{m_{k}}}{m_{k}!}e^{-\lambda_{T}}$$

and the PCRLB can be calculated recursively by the EIRF method described in Section 4.2. The overall measurement contribution (given in (29)) is then:

(44) $$J_{Z}\left(k\right)=E\left[m_{k}\right]J_{Z1}\left(k\right)=\lambda_{T}J_{Z1}\left(k\right)$$

where:

(45) $$J_{Z1}\left(k\right)=E_{X_{k}}\left[H_{k}^{T}R_{k}^{-1}H_{k}\right]$$

and the expression for *H_k_* is a 2*4 sub-matrix of $H_{k}^{e}$ given in Section 4.1:

(46) $$H_{k}\left[\begin{array}{llll} \frac{X_{k}\left(1\right)}{\sqrt{{\left[X_{k}\left(1\right)\right]}^{2}+{\left[X_{k}\left(3\right)\right]}^{2}}} & 0 & \frac{X_{k}\left(3\right)}{\sqrt{{\left[X_{k}\left(1\right)\right]}^{2}+{\left[X_{k}\left(3\right)\right]}^{2}}} & 0\\ \frac{-X_{k}\left(3\right)}{{\left[X_{k}\left(1\right)\right]}^{2}+{\left[X_{k}\left(3\right)\right]}^{2}} & 0 & \frac{X_{k}\left(1\right)}{{\left[X_{k}\left(1\right)\right]}^{2}+{\left[X_{k}\left(3\right)\right]}^{2}} & 0 \end{array}\right]$$

The method of calculating the mathematical expectation with respect to the state vector in (45) is the same as that described in Example 1.

**Figure 5.**
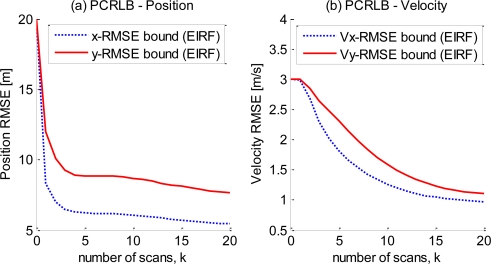
The $\sqrt{\text{PCRLB}}$ curves of the multi-measurement extended target tracking for (a) the target position in the directions of the *x* and *y* axes and (b) the target velocity in the directions of the *x* and *y* axes.

**Figure 6.**
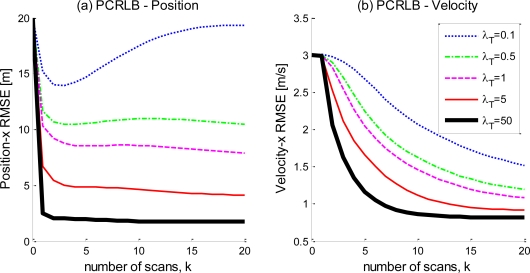
The PCRLB of the multi-measurement extended target tracking for different mean numbers of measurements for (a) the target position in the direction of the *x* axis. (b) and the target velocity in the direction of the *x* axis.

In the simulation, the sensor is also static and at the origin of the coordinate system, while the target moves with initial velocity (*v_x_*_0_, *v_y_*_0_) = (10, 15) starting from the initial position (15000,10000). The initial FIM is *J*_0_ = {[*diag* (20, 3, 20, 3)]^−2^}^−1^. The covariance of the state noise is *Q_k_* = *diag* (3, 0.1, 3, 0.1), and the measurement noise is zero-mean white Gaussian noise with standard deviations of *σ_ρ_* = 5 and *σ_θ_* = 0.1°. The mean number of measurements per frame is *λ_T_* = 2.5, and the time interval is *T* = 1. All the parameter units are in the metric system. The estimation bounds calculated by the EIRF approach for the position and velocity along both the *x* and *y* axes are shown in Figure 5. Equation (28) indicates that the estimation performance bound is affected by the number of measurements originating from the target. In the EIRF methodology, the overall impact is manifested as a constant scalar, the mean number of measurements per frame. The $\sqrt{\text{PCRLB}}$ curves (calculated by the EIRF approach) for the target position and velocity in the direction of the *x* axis for various values of the Poisson intensity (the mean number of measurements per frame) *λ_T_* is shown in Figure 6(a) and (b), respectively. The curve corresponding to *λ_T_* = 1 is equivalent to the point target tracking bound, and, therefore, the simulation results indicate that the large quantity of measurements decreases the estimation bound, therefore possibly improving the estimation accuracy.

## 5. Conclusions

In this article, the calculation of the PCRLB for two types of extended target tracking models is reported. For the equation extension (first type) extended target model, the dynamic and measurement equations are extensions of those of the point target models and are still in the general nonlinear filtering form. The PCRLB is then calculated through the recursive formulation, and the bound of the target centroid dynamics estimation of the extended model is always smaller than that of the point model. For the spatial distribution (second type) extended target model, the general form of the measurement contribution for a specific number of measurements with no clutter is presented in the paper, and the EIRF approach is introduced to calculate the overall measurement contribution and therefore the PCRLB. Illustrative simulation examples for the two types of extended target tracking models are also presented to verify the theoretical development and demonstrate the influence of parameters on the PCRLB. The theoretical and numerical results suggest the superior performance bound for both the two types of extended target models.
